# Deep-Learning-Based Thrombus Localization and Segmentation in Patients with Posterior Circulation Stroke

**DOI:** 10.3390/diagnostics12061400

**Published:** 2022-06-06

**Authors:** Riaan Zoetmulder, Agnetha A. E. Bruggeman, Ivana Išgum, Efstratios Gavves, Charles B. L. M. Majoie, Ludo F. M. Beenen, Diederik W. J. Dippel, Nikkie Boodt, Sanne J. den Hartog, Pieter J. van Doormaal, Sandra A. P. Cornelissen, Yvo B. W. E. M. Roos, Josje Brouwer, Wouter J. Schonewille, Anne F. V. Pirson, Wim H. van Zwam, Christiaan van der Leij, Rutger J. B. Brans, Adriaan C. G. M. van Es, Henk A. Marquering

**Affiliations:** 1 Department of Biomedical Engineering and Physics, Amsterdam University Medical Centers, Location AMC, 1105 AZ Amsterdam, The Netherlands; i.isgum@amsterdamumc.nl (I.I.); h.a.marquering@amsterdamumc.nl (H.A.M.); 2 Department of Radiology and Nuclear Medicine, Amsterdam University Medical Centers, Location AMC, 1105 AZ Amsterdam, The Netherlands; a.e.bruggeman@amsterdamumc.nl (A.A.E.B.); c.b.majoie@amsterdamumc.nl (C.B.L.M.M.); l.f.beenen@amsterdamumc.nl (L.F.M.B.); 3 Informatics Institute, University of Amsterdam, 1012 WX Amsterdam, The Netherlands; e.gavves@uva.nl; 4 Department of Neurology, Erasmus MC, University Medical Center, 3015 GD Rotterdam, The Netherlands; d.dippel@erasmusmc.nl (D.W.J.D.); n.boodt@erasmusmc.nl (N.B.); s.denhartog@erasmusmc.nl (S.J.d.H.); 5 Department of Radiology & Nuclear Medicine, Erasmus MC, University Medical Center Rotterdam, 3015 GD Rotterdam, The Netherlands; p.j.vandoormaal@erasmusmc.nl (P.J.v.D.); s.cornelissen@erasmusmc.nl (S.A.P.C.); 6 Department of Public Health, Erasmus MC, University Medical Center Rotterdam, 3015 GD Rotterdam, The Netherlands; 7 Department of Neurology, Amsterdam University Medical Centers, Location AMC, 1105 AZ Amsterdam, The Netherlands; y.b.roos@amsterdamumc.nl (Y.B.W.E.M.R.); j.brouwer@amsterdamumc.nl (J.B.); 8 St. Antonius Hospital, 3435 CM Nieuwegein, The Netherlands; w.schonewille@antoniusziekenhuis.nl; 9 Department of Neurology, Maastricht University Medical Center, School for Cardiovascular Diseases (CARIM), 6229 ER Maastricht, The Netherlands; fav.pirson@mumc.nl; 10 Department of Radiology and Nuclear Medicine, Maastricht University Medical Center, School for Cardiovascular Diseases (CARIM), 6229 ER Maastricht, The Netherlands; w.van.zwam@mumc.nl (W.H.v.Z.); christiaan.vander.leij@mumc.nl (C.v.d.L.); rutger.brans@mumc.nl (R.J.B.B.); 11 Department of Radiology and Nuclear Medicine, Leiden University Medical Center, 2333 ZA Leiden, The Netherlands; a.c.g.m.van_es@lumc.nl

**Keywords:** posterior stroke, deep learning, CTA, NCCT, thrombus, localization, segmentation

## Abstract

Thrombus volume in posterior circulation stroke (PCS) has been associated with outcome, through recanalization. Manual thrombus segmentation is impractical for large scale analysis of image characteristics. Hence, in this study we develop the first automatic method for thrombus localization and segmentation on CT in patients with PCS. In this multi-center retrospective study, 187 patients with PCS from the MR CLEAN Registry were included. We developed a convolutional neural network (CNN) that segments thrombi and restricts the volume-of-interest (VOI) to the brainstem (Polar-UNet). Furthermore, we reduced false positive localization by removing small-volume objects, referred to as volume-based removal (VBR). Polar-UNet is benchmarked against a CNN that does not restrict the VOI (BL-UNet). Performance metrics included the intra-class correlation coefficient (ICC) between automated and manually segmented thrombus volumes, the thrombus localization precision and recall, and the Dice coefficient. The majority of the thrombi were localized. Without VBR, Polar-UNet achieved a thrombus localization recall of 0.82, versus 0.78 achieved by BL-UNet. This high recall was accompanied by a low precision of 0.14 and 0.09. VBR improved precision to 0.65 and 0.56 for Polar-UNet and BL-UNet, respectively, with a small reduction in recall to 0.75 and 0.69. The Dice coefficient achieved by Polar-UNet was 0.44, versus 0.38 achieved by BL-UNet with VBR. Both methods achieved ICCs of 0.41 (95% CI: 0.27–0.54). Restricting the VOI to the brainstem improved the thrombus localization precision, recall, and segmentation overlap compared to the benchmark. VBR improved thrombus localization precision but lowered recall.

## 1. Introduction

Stroke due to a large vessel occlusion in the posterior circulation accounts for approximately 1% [1,2] of all cases of acute ischemic stroke and is associated with poor outcome [3]. Posterior circulation stroke (PCS) may have an acute onset or a progressive and stuttering onset, and may produce symptoms that are not typically associated with anterior stroke, such as vertigo and nausea [1]. Therefore, PCS is associated with a higher chance of misinterpretation and under-diagnosis in clinical practice than anterior circulation stroke (ACS) [4,5], which has resulted in long delays in door-to-needle times of patients suffering from a PCS compared to patients suffering from an ACS [6,7]. Reducing time from symptom onset to treatment may improve the outcome of patients suffering from a PCS who are treated with intravenous alteplase treatment and/or endovascular treatment [8,9,10].

Localizing an occluding thrombus causing PCS on radiological imaging is not a problem for expert neuro-radiologists and can be done quickly [11]. However, timely access to the services of an expert neuro-radiologist may not be possible in primary stroke centers because of the limited number of available neuro-radiologists. Automated localization of PCS may help to avoid misinterpretation in the case of a PCS. In addition to localization, segmentation of the thrombus would allow for automatic quantification of thrombus volume. Thrombus volume was previously reported to be negatively associated with recanalization and a higher likelihood of poor functional outcome [12,13]. 

Recently proposed methods for the automated localization and segmentation of thrombi in stroke patients make use of convolutional neural networks (CNNs) [14,15,16]. Training CNNs requires imaging data from a large number of patients to reach an acceptable localization accuracy. For anterior circulation stroke, data from large numbers of patients are available even in single medical centers. However, data from patients suffering from a PCS are scarce. 

As opposed to thrombi in patients with an ACS, thrombi in patients with a PCS occur in a more limited area around the brainstem. This characteristic can be used to improve the performance of a CNN-based thrombus localization and segmentation method. A CNN can learn how to center a moving volume-of-interest (VOI) on the brainstem and evaluate only locations with a high likelihood of a thrombus. We hypothesize that tracking the brainstem with a small VOI improves the localization and segmentation of thrombi in the posterior circulation. In addition, we hypothesize that by removing small, segmented thrombi, the number of false positive thrombus localizations can be reduced.

In this study, we aim to develop and evaluate the first automatic CNN-based method for thrombus localization and segmentation on baseline on non-contrast CT (NCCT) and CT angiography (CTA) in patients suffering from a PCS.

## 2. Materials and Methods

### 2.1. Materials

The data used in this study were obtained from a prospective and nationwide observational study on endovascular treatment in the Netherlands (the MR CLEAN Registry [17]). The data were collected in 16 centers that performed endovascular treatment in the Netherlands between March 2014 and January 2019. The dataset used in our study included all patients from the MR CLEAN Registry that suffered from a PCS and consisted of NCCT and CTA scans from 268 patients. Hence, patients were also included if they suffered from an anterior circulation stroke (three patients) or a subarachnoid hemorrhage (one patient) in addition to a PCS. Patients were excluded if no baseline CTA was available, the scan quality was too low, registration of the NCCT to the CTA was unsuccessful, or if the patient had a non-occlusive thrombus or dissection. The Medical Ethics Committee of the Erasmus University Medical Center in Rotterdam, the Netherlands, approved the MR CLEAN Registry (MEC-2014-235). In addition, the institutional review board of each participating center approved the MR CLEAN research protocol.

### 2.2. Reference Annotations

Two types of annotations were made: reference thrombus segmentations, and annotations of the reference path, which runs through the structures of the brainstem. The reference segmentations of the thrombi were obtained by manual annotation. One trained observer (RZ) manually segmented the thrombi, and the second observer (AAEB) corrected the segmentations if necessary. A window width of 30 Hounsfield units (HU) and a center level of 35 HU was used for the NCCT scan, and a window width of 600 HU and a center level of 300 HU was used for the CTA scan. Annotations were made using ITK-Snap. For each patient, a case record form was available from an imaging core lab, which indicated the location of the occlusion and whether a hyperdense artery sign (HAS) was visible on the NCCT. The information about the location of the occlusion was used to guide the manual annotation. If the patient had a HAS, the NCCT was used to create the reference segmentation. Otherwise, the absence of arterial filling on the CTA was used to create the reference segmentation. 

The reference path through the brainstem was annotated by one trained observer (RZ). The path started in the spinal cord, continued through the medulla oblongata, pons and midbrain. The reference path was marked by annotating a single voxel per axial slice in ITK Snap. 

### 2.3. Preprocessing

To limit the size of the scan, all slices 25 cm below the top of the skull were excluded. Next, to align the anatomical structures in the CTA to those in the NCCT, the CTAs were registered to the NCCT by using rigid transformations. Finally, the voxel intensities were clipped between −100 and 200 HU and normalized between minus one and one. The preprocessing was done using SimpleITK and python 3.8.5.

### 2.4. CNNs for Automatic Posterior Circulation Thrombus Localization and Segmentation

Existing deep-learning methods result in false positive localization and segmentation if they are used on small objects. We developed a method to reduce the number of false positive localizations by restricting the VOI to a region that included the brain stem, which was most likely to contain a thrombus in patients suffering from a PCS. To restrict the VOI to the brainstem, the method had to learn how to move the VOI towards the brainstem on an axial slice. The movement from the center of the VOI at a given location was parameterized as a circle with a radius and an angle (polar coordinates). The method learned to regress these polar coordinates and was named polar-UNet. In addition to regressing the polar coordinates, polar-UNet classified what action should be taken at each step: to move the VOI within the current axial slice, segment and move up one axial slice, or to stop the inference procedure. 

**Figure 1:**
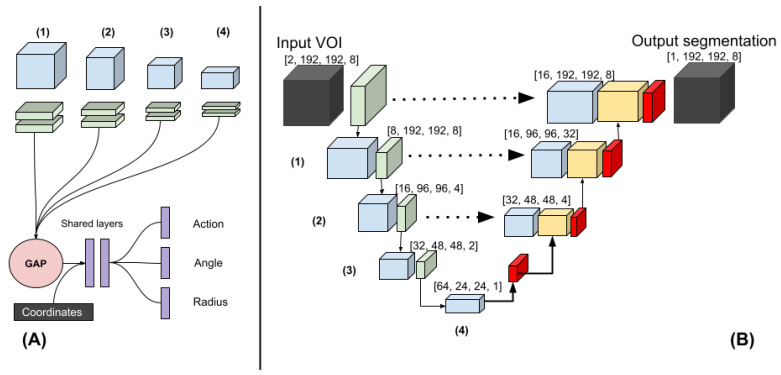
(**A**) The polar-UNet is created by attaching the following operations to the BL-UNet. The features from four levels in the down-sampling path are input to four blocks consisting of two convolutions. The output is global average-pooled and concatenated before being passed to the fully connected shared layers. Finally, individual fully connected layers are used to classify the action and regress the angle and the radius. (**B**) Three-dimensional Baseline UNet (BL-UNet). Three-dimensional ResNet blocks followed by a max-pooling operation (green) were used to construct the down-sampling path (**left**). Three-dimensional ResNet blocks followed by a transposed convolution were used to construct the up-sampling path (**right**). The features generated in the down- and up-sampling paths are blue and yellow, respectively. Skip connections were added between the up-sampling and down-sampling paths.

The architecture of the regression and classification heads of the polar-UNet is shown in Figure 1A. To create the polar-UNet, the regression and classification head were added to the down-sampling path of the baseline UNet (BL-UNet), which is shown in Figure 1B. Features were extracted at four levels of the down-sampling path. These features were input into four separate convolutional layers and global average-pooled. Next, the extracted features were concatenated with the relative coordinates of the VOI in the scan volume and input into two shared fully connected layers, which consisted of 256 neurons each. The output of the shared layer was passed to the individual network heads, which regressed either the angle or the radius, or classified the action. The angle regression head used a TanH activation, the radius regression head used a sigmoid activation, and the action classification head used the softmax activation function in the output layer. Finally, polar-UNet also had the same up-sampling path as BL-UNet.

The BL-UNet was inspired by U-Net [18] and consisted of 3D ResNet [19] blocks. The down-sampling path started with two convolutional layers, which consisted of a kernel size of three and a stride of one. It was followed by a max-pooling operation with a kernel size and stride of two. Subsequently, three down-sampling blocks, which consisted of a 3D ResNet layer each, were added. The first two blocks were followed by a max-pooling layer with a stride and pooling size of two. The up-sampling path started with a transposed convolution. Next, two up-sampling blocks, which consisted of a ResNet block followed by a transposed convolution, were added. Each transposed convolution had a stride of two and a kernel size of three. The features of the down-sampling path were concatenated with the features in the up-sampling path. The up-sampling path was followed by a three-by-three convolution and a one-by-one convolution. During inference, BL-UNet was applied to a non-overlapping grid of VOIs extracted from the entirety of each scan. The CNNs were implemented using Pytorch 1.5.1 [20].

### 2.5. Experimental Setup

The BL-UNet was randomly initialized and trained on VOIs sampled from the scan volumes. To ensure the data contained sufficient positive examples, 40 percent of the sampled VOIs contained a thrombus. The up-sampling and down-sampling paths of the polar-UNet were initialized by reusing the weights obtained by the pre-trained BL-UNet and were not updated during training. The BL-UNet used group normalization with 4 groups, the ReLU activation, a batch size of 32, a cyclical learning rate schedule, the Adam optimizer and a weight decay of $20^{-6}$. The BL-UNet was trained for 300 epochs with 148 iterations per epoch. 

The polar-UNet applied batch-normalization to the layers that were updated during training and used a batch size of 128. The focal loss function was used for the segmentation and classification tasks, and the $L_{2}$ loss [21] for the regression tasks. The polar-UNet was trained for 100 epochs with 148 iterations per epoch. Both CNNs used a maximum learning rate of $10^{-3}$, a minimum learning rate of $20^{-5}$, and the weight decay was set to $20^{-5}$. The learning rate was linearly increased to the maximum value in 300 iterations and decreased to the minimum value in 300 iterations. 

The polar coordinate reference values that were needed to move the VOI to the area around the brainstem were calculated using the reference path. The angle reference value was calculated as the angle between the coordinates of the center of the VOI and the coordinates of the reference path on the same axial slice. To allow for easier optimization, the angle reference values were normalized between 0 and 2. The radius reference value indicated the step size that the VOI had to be moved to minimize the Euclidean distance between the center of the VOI and the reference path on the same axial slice. The radius was limited to the width of the VOI and normalized between 0 and 1 to allow for easier optimization. 

The inference procedure of the polar-UNet is shown in the Supplementary Materials Algorithm S1. We initialized the VOI at the center of the most caudal axial slice in the scan. Per axial slice, the VOI could only be moved within the axial slice 10 times before the VOI location was moved up by one axial slice. If the VOI moved out of the bounds of the volume, the VOI location was moved up by one slice and reset to the center of the axial slice. 

Data augmentation was applied during training. The axial slices in the scan volumes were rotated at an angle between minus ten and ten degrees and, with a probability of 0.5, were flipped. Furthermore, the scan volumes were magnified by a factor between 0.9 and 1.1 and translated in the axial plane by a maximum of 80 voxels. Polar coordinates reference values were updated accordingly. The sampled VOIs had dimensions of 192 × 192 × 8. 

We used stratified five-fold cross-validation to evaluate the performance of the CNN architectures. The hyper-parameters used in our study were found by evaluating multiple sets of hyper-parameters on the first fold and selecting the ones with the highest testing performance. To prevent inflation of the results due to the first fold being used to find the optimal hyper-parameters, this fold was excluded from the evaluation. 

To improve the localization precision of the CNNs, only connected components larger than 0.065 mL were included in further analyses. We refer to this step as volume-based removal (VBR). 

### 2.6. Evaluation

To evaluate the localization performance of our models, we calculated the thrombus localization precision and recall. Hence, the number of true positive (TP), false positive (FP), and false negative (FN) localizations had to be calculated. To calculate the TP, FP, and FN localizations, the segmentation maps had to be divided into individual connected regions. Thus, a connected-components analysis was run on the automatically created and manually annotated segmentations. If two connected components from the manually annotated segmentation and the corresponding automatically created segmentation had ten percent or more of their voxels overlapping, this was counted as a TP localization. If a connected component from the automatically created segmentation had less than ten percent overlap with a connected component from the manually annotated segmentation it was counted as a FP localization. If a connected component from the manually annotated segmentation did not have more than ten percent overlap with any of the connected components from the automatically created segmentation, it was counted as a FN. 

The intra-class correlation coefficient (ICC) was used to evaluate the agreement between the volume derived from the automatically and manually segmented thrombi. We included the 95% confidence interval (95% CI) in our analysis. Furthermore, we performed a Bland-Altman analysis to evaluate the bias and limits of agreement of the volume measurements. We tested whether the ICC of the automated segmentations created by the two CNN architectures with and without VBR differed significantly by using Fishers r to z transformation.

The segmentation overlap of the CNNs was evaluated by calculating the Dice coefficient between the reference and the automatic segmentation. If no thrombus was visible in the scan, the Dice coefficient would always equal zero and would always deflate the results artificially. Hence, patients with no visible thrombus in the scan were excluded from the analysis of the Dice coefficient. 

The obtained metrics were tested for normality with Shapiro-Wilk tests. If the obtained metrics followed a normal distribution, paired *t*-tests were used for comparison, otherwise Wilcoxon rank-sum tests were used. A Bonferroni correction was applied to P-values to correct for family-wise error. Finally, thrombi in the posterior circulation most commonly occur in the basilar artery. Therefore, we evaluated whether thrombus location influenced true positive thrombus localization, volume agreement, and segmentation overlap. Thrombus location was obtained from the case report file of each patient. The Python library Pingouin, version 0.3.1 was used for all statistical testing.

## 3. Results

The patient flow chart is shown in the Supplementary Materials Figure S1. In total, 187 patients were included in our study. The baseline characteristics of the included patients are shown in the Supplementary Materials Table S1.

In 185 out of 187 scans (99%) a thrombus was visible and was manually annotated. After exclusion of the patients in the test set of the first fold, due to this fold being used for hyper-parameter optimization, 149 scans were left to evaluate the results on. 

The thrombus localization recall varied between 129 (69%) and 146 (82%). The thrombus localization recall before and after VBR for the BL-UNet and polar-UNet is shown in Table 1. Overall, the Polar-UNet improved the thrombus localization recall over BL-UNet, and VBR reduced the thrombus localization recall of both CNNs.

**Figure 2:**
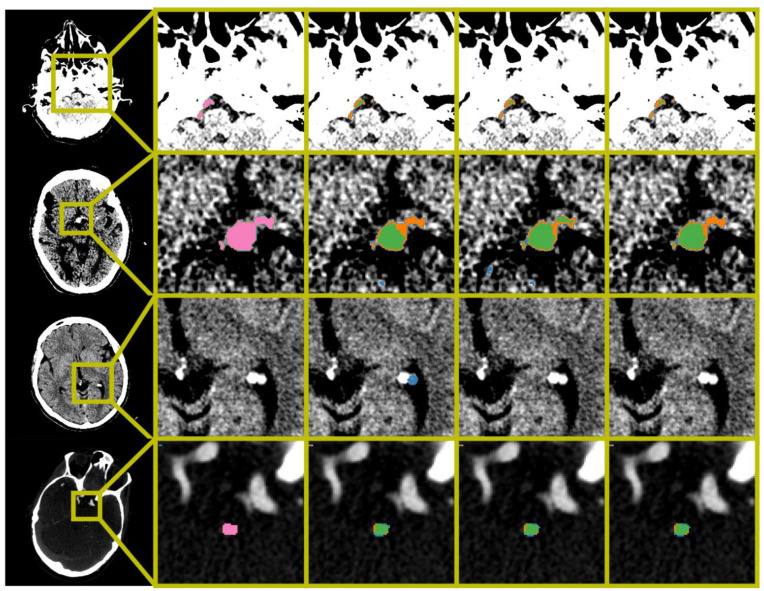
Examples of automatic segmentation results obtained by BL-UNet and Polar-UNet. From the left to right column: The original scan with a bounding box indicating the zoom location, the ground truth segmentation map, the results obtained from the BL-UNet without volume-based removal (VBR), the results obtained from the Polar-UNet without VBR, and the results obtained from the Polar-UNet with VBR. The top three rows display NCCT scans; the bottom row shows a CTA scan. The top row shows the difficulty all CNN methods have with segmenting a thrombus in the vertebral arteries. The second row from the top shows an example of small false positives removed by the VBR step. The third row from the top row shows false positives that are removed by restricting the volume-of-interest to the posterior circulation with Polar-UNet. The bottom row shows an example of a scan without a hyperdense artery sign. The segmentation maps show the ground truth (pink), true positive (green), false negative (orange) and false positive (blue). The NCCT scans were plotted using a window center level of 35, with a window width of 30. The CTA scan was plotted using a window center level of 300, with a window width of 600.

The thrombus localization precision before and after VBR for the BL-UNet and polar-UNet is shown in Table 1. In all cases, Polar-UNet improved the thrombus localization precision over the BL-UNet, and VBR improved the thrombus localization precision of both CNNs (Figure 2). However, for all cases the thrombus localization precision is low with an overall maximum of 62%.

**Figure 3:**
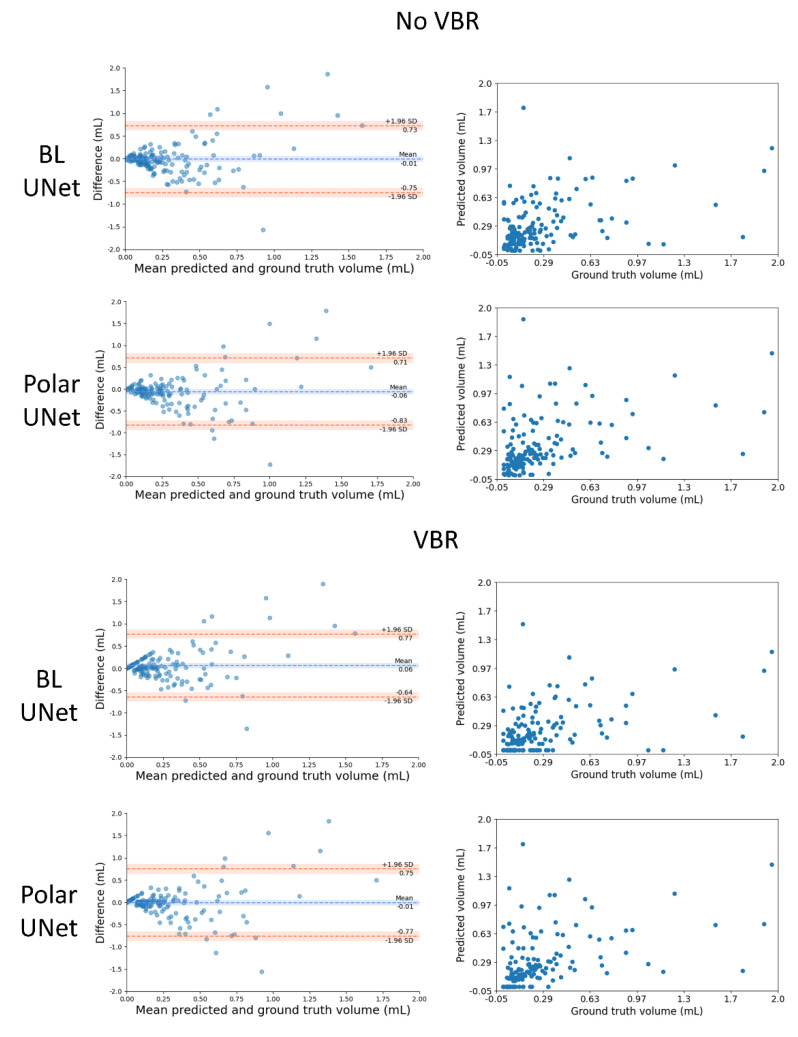
Comparison of the automated and manually segmented volume for the BL-UNet and Polar-UNet with and without volume-based removal (VBR). Left column: Bland–Altman plots of the lesion volumes. The volumes corresponding to the reference and automatic segmentations are shown on the x-axis, and the volume difference is shown on the y-axis. Right column: scatter plots comparing lesion volumes derived from the reference segmentations (y-axis) and from the automatic segmentations determined by the CNN (x-axis).

The median thrombus volume was 0.15 (IQR: 0.07–0.34) mL. The ICC for the volume agreement of the BL-UNet and Polar-UNet without VBR were 0.38 (95% CI: 0.23–0.51) and 0.4 (95% CI: 0.25–0.53). With VBR, the ICCs for the volume agreement of the BL-UNet and Polar-UNet were both 0.41 (95% CI: 0.27–0.54). There was no statistically significant difference between the volumetric agreement of the various methods. The Bland-Altman analysis for each of the CNNs resulted in biases ranging from −0.06 mL for the Polar-UNet without VBR to 0.06 mL for the BL-UNet with VBR. The LoAs were the smallest for the BL-UNet with VBR, ranging from −0.64 to 0.77 mL. The LoAs were largest for the Polar-UNet without VBR, ranging from −0.83 to 0.71 mL. The bias and LoAs for the volume analysis are shown in the Supplementary Materials Table S3, and the Bland-Altman results are shown in Figure 3. 

The Dice coefficients for the automated thrombus segmentation are shown in Table 1. The results of the Wilcoxon rank-sum test are shown in the Supplementary Materials Table S2. The results show that the Polar-UNet with VBR results in a statistically significantly larger Dice coefficient than the other methods. Furthermore, the Polar-UNet without VBR results in a significantly greater Dice coefficient than the BL-UNet with or without VBR. 

## 4. Discussion

In this study we have presented the first CNN-based method to segment and localize thrombi in patients suffering from PCS. The restriction of the VOI to areas in the vicinity of the brainstem improved the segmentation overlap and thrombus localization precision and recall over the baseline method. The VBR of small objects improved the thrombus localization precision of our method but reduced the recall. Regardless of whether VBR of small objects is applied, the bias of the automated volume quantifications is small, but the limits of agreement are large. Finally, there is room to improve the methods volume agreement and segmentation overlap.

Our study is the first to address the automated localization and segmentation of thrombi in the posterior circulation on CTA and NCCT. Prior work has focused on identifying scans with a large vessel occlusion (LVO) in the anterior circulation on a single-phase [22,23] and multi-phase [14,24] CTA. Two studies included patients suffering from both types of stroke, either in the posterior or anterior circulation [14,25]. However, a limitation is that not all studies report results specific to the posterior stroke patient group [14]. Another limitation of prior work is that the thrombus is not localized, nor is it segmented. Only the presence or absence of an LVO in a scan is indicated.

Other work aimed to localize or segment thrombi on NCCT scans of patients suffering from either anterior or posterior circulation stroke [15,26,27]. One study used feature extraction and a random forest classifier [27]. This study did not mention the location of the thrombi in their dataset. Other work has used deep learning to localize or segment thrombi on NCCT scans of patients in the anterior circulation [15,26]. A key feature of these methods is that a comparison between hemispheres was made to improve localization or segmentation performance. A comparison between hemispheres has no added value to PCS thrombus localization and segmentation, because of the absence of lateral symmetry of the basilar artery.

Our study has several limitations. First, our method is not accurate at localizing and segmenting thrombi in the vertebral and posterior cerebral artery. This is likely because of the rareness of thrombi in these sections. Hence, data from few patients with occlusions in these regions were available to train our method on. In contrast, our method does achieve a good localization precision and a reasonable segmentation accuracy for thrombi in the basilar artery, which is the most common location for PCS. Second, our data was annotated by one trained observer. Hence, the inter-observer agreement could not be assessed. However, the manual segmentations were verified and corrected by another experienced observer. Third, the method achieved a low volumetric agreement and segmentation overlap between the manually and automatically segmented thrombi. Still, the volumetric agreement is similar to those reported in a study that focuses on thrombus segmentation and localization in the anterior circulation [15]. This is despite our method not being able to use lateral symmetry information, as was done by methods for anterior stroke localization and segmentation. Furthermore, the low Dice coefficient can be explained by the small size of the thrombi. For small object segmentation, the Dice coefficient is over-sensitive to small errors [28]. Fourth, manual segmentation of thrombi in patients without a HAS and with poor collateral status on single phase CTA leads to an overestimation of thrombus length [29]. The overestimation of thrombus length is caused by a delayed contrast arrival. Hence, our method may, just like manual segmentations, overestimate the true length of the occluding thrombus. Fifth, since there were no additional large datasets with patients with posterior stroke available, our method lacks validation on an external dataset. Thus, results may vary when our algorithm is trained on or applied to other datasets. However, the data in our study were collected from sixteen different centers. Our algorithm was nonetheless evaluated on a heterogeneous set of scans. As more image data of patients with posterior stroke become available, our method may be improved and validated by including this image data.

A valuable application of deep learning is to automate time-critical and labor-intensive tasks such as the localization and segmentation of small objects in radiological imaging. Our study has shown that for small object segmentation and localization, a standard deep-learning-based segmentation method would result in large amounts of false positives. In this study we have presented an approach that combines restricting the segmentation and localization to the region in which the occlusion occurs, and excluding small objects. We have shown that this combination reduces the number of false positives.

The automated posterior thrombus localization and segmentation method is the first step towards a method that allows associations between thrombus characteristics, such as thrombus volume and length, and PCS outcome to be easily studied in clinical trials.

## Figures and Tables

**Table 1:** Thrombus localization recall and precision in the posterior circulation and the Dice coefficient of the BL-UNet and Polar-UNet, with and without volume-based removal (VBR). Results are shown for the entire dataset (overall), the vertebral artery (VA), basilar artery (BA), posterior cerebral artery (PCA), thrombi which crossed multiple segments (VA + BA, BA + PCA, VA + BA + PCA), and patients for whom the thrombus location was not indicated (NAV). * Two scans did not have a visible thrombus. Hence, the Dice coefficient was calculated for 8 patients with a thrombus in the VA and 4 with an unspecified occlusion location (NAV).

Localization Recall		Overall N = 149	VA N = 9	BA N = 37	VA + BA N = 22	BA + PCA N = 49	VA + BA + PCA N = 8	PCA N = 19	NAV N = 5
**No VBR**	**BL-UNet**	0.78	0.58	0.86	0.76	0.89	1	0.46	0
	**Polar-Unet**	0.82	0.75	0.94	0.83	0.87	0.9	0.46	0.33
**VBR**	**BL-UNet**	0.69	0.42	0.61	0.69	0.73	0.8	0.15	0
	**Polar-UNet**	0.75	0.67	0.81	0.83	0.8	0.9	0.31	0.33
**Localization Precision**									
**No VBR**	**BL-UNet**	0.09	0.10	0.09	0.12	0.11	0.15	0.04	0
	**Polar-UNet**	0.14	0.27	0.14	0.17	0.13	0.23	0.05	0.04
**VBR**	**BL-UNet**	0.56	0.56	0.55	0.65	0.53	0.73	0.29	0
	**Polar-UNet**	0.62	0.57	0.66	0.77	0.55	1	0.4	0.2
**Dice Coefficient**		N = 147 *							
**No VBR**	**BL-UNet**	0.38	0.30	0.42	0.42	0.45	0.5	0.15	0.09
	**Polar-UNet**	0.44	0.34	0.49	0.50	0.48	0.56	0.2	0.26
**VBR**	**BL-UNet**	0.35	0.21	0.37	0.42	0.44	0.5	0.07	0
	**Polar-UNet**	0.44	0.33	0.50	0.52	0.48	0.62	0.12	0.17

## Data Availability

A Docker container to run the algorithm is available upon reasonable request.

## Supplementary Materials

The following supporting information can be downloaded at: https://www.mdpi.com/article/10.3390/diagnostics12061400/s1, Video S1: thrombus segmentation using Polar-UNet, Figure S1. Flow chart showing the inclusion of the patients with a posterior circulation stroke in the MR CLEAN Registry. Exclusion criteria were: no CTA available (n = 21), poor image quality (n = 16), preprocessing error (n = 42), or a non-occlusive thrombus or dissection (n = 2). In total, 187 patients were included, Table S1. Baseline characteristics, treatment, and time data for included patients with posterior circulation stroke, Table S2. Wilcoxon rank-sum test on pairwise differences between the Dice coefficient and the bias of the volume differences. The W-statistic and *p*-value are shown. The Polar UNet (with VBR) produced a significantly greater Dice coefficient relative to the other methods. The Polar UNet produced a significantly greater Dice coefficient relative to the BL-UNet (with VBR) and the BL-UNet. No method significantly improved the bias of the volume estimation other than the Po-lar-UNet (with VBR) over the Polar-UNet, Table S3. Bias and limits of agreement between the automatically and manually segmented thrombus volumes, respectively, for the BL-UNet and Polar-UNet with and without volume-based removal (VBR). Algorithm S1. Inference procedure for the Polar U-Net.
