# Supplementary material for: Deep-Learning-Based Thrombus Localization and Segmentation in Patients with Posterior Circulation Stroke
Source: Diagnostics (Basel). 2022 Jun 6;12(6):1400. doi: 10.3390/diagnostics12061400 (PMC9222185; doi:10.3390/diagnostics12061400)
Supplement: Supplementary file 1 [file diagnostics-12-01400-s001.zip › diagnostics-1741078_supplementary materials.pdf]

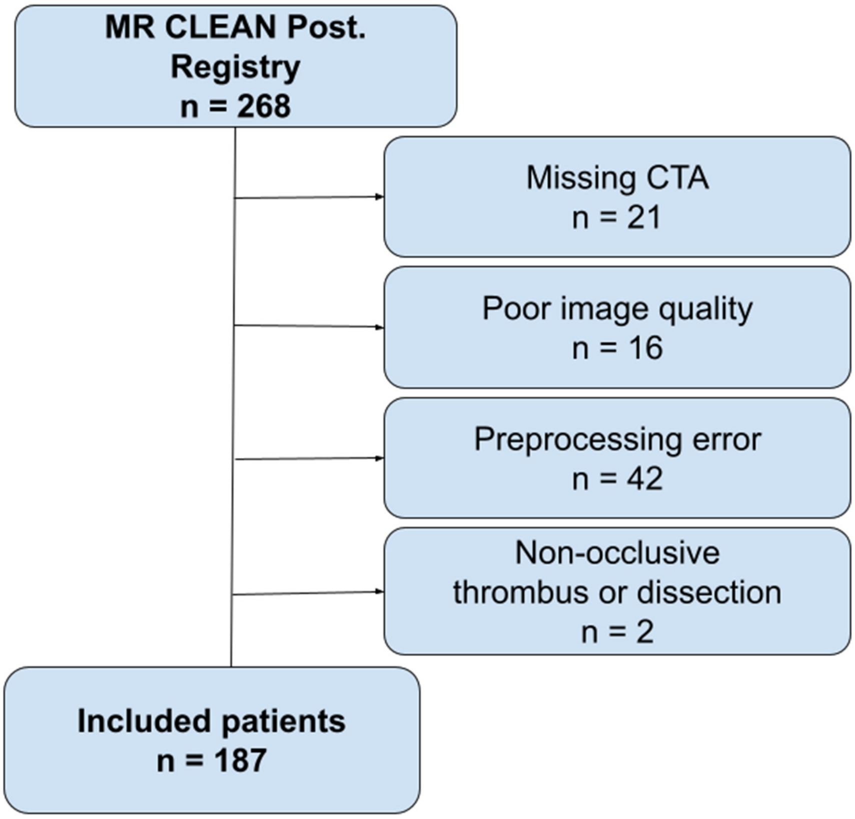

Figure S1. Flow chart showing the inclusion of the patients with a posterior circulation stroke in the MR CLEAN Registry. Exclusion criteria were: no CTA available (n = 21), poor image quality (n = 16), preprocessing error (n = 42), or a non-occlusive thrombus or dissection (n = 2). In total, 187 patients were included.

Table S1. Baseline characteristics, treatment, and time data for included patients with posterior circulation stroke.

Parameter	Posterior Stroke
Clinical	
Age, years, median (IQR)	65.5 (20.8)
Sex, F, no. [%]	72/186 [38.7]
NIHSS at baseline, median(IQR) [N]	15 [22] (183)
Prior Conditions	
Diabetes mellitus, no. [%]	32/184 [17.4]
Hypertension, no. [%]	97/181 [53.6]
Stroke, no. [%]	30/183 [16.4]
Atrial fibrillation, no. [%]	25/184 [13.6]
Pre-Stroke mRS	
0, no. [%]	126/180 [70]
1, no. [%]	19/180 [10.6]
2, no. [%]	17/180 [8.4]
3, no. [%]	7/180 [3.9]
4, no. [%]	8/180 [4.4]

5, no. [%]	3/180 [1.7]
Treatment	
IV Thrombolysis, no. [%]	102/186 [54.8]
Time	
Stroke onset to IVT, minutes, median [IQR](N)	98 [73] (93)

Table S2. Wilcoxon rank-sum test on pairwise differences between the Dice coefficient and the bias of the volume differences. The W-statistic and p-value are shown. The Polar UNet (with VBR) produced a significantly greater Dice coefficient relative to the other methods. The Polar UNet produced a significantly greater Dice coefficient relative to the BL-UNet (with VBR) and the BL-UNet. No method significantly improved the bias of the volume estimation other than the Polar-UNet (with VBR) over the Polar-UNet.

**Method 1**	**Method 2**	**Dice Coefficient**		**Bias**	
		**W**	***p*-Value**	**W**	***p*-Value**
BL UNet	BL UNet (with VBR)	3039	>0.05	3698	>0.05
BL UNet	Polar Unet	2415	<0.01	5392	>0.05
BL UNet	Polar Unet (with VBR)	2481	<0.01	4361	>0.05
BL UNet (with VBR)	Polar Unet	2551	<0.01	4832	>0.05
BL UNet (with VBR)	Polar Unet (with VBR)	1367	<0.01	3703	>0.05
Polar Unet	Polar Unet (with VBR)	2431	<0.05	3153	<0.01

Table S3. Bias and limits of agreement between the automatically and manually segmented thrombus volumes, respectively, for the BL-UNet and Polar-UNet with and without volume-based removal (VBR).

		**Bias**	**LoA**
**No VBR**	**BL-UNet**	−0.01 mL	−0.75 to 0.73 mL
	**Polar-UNet**	−0.06 mL	−0.83 to 0.71 mL
**VBR**	**BL-UNet**	0.06 mL	−0.64 to 0.77 mL
	**Polar-Unet**	−0.01 mL	−0.77 to 0.75 mL

Algorithm S1. Inference procedure for the Polar U-Net.

```
Input: scan, max_iterations_per_slice
Initialize: starting_coordinates, scan_prediction
for slice_number in number_slices:
    for iteration in max_iterations_per_slice:
        Extract voi from scan given starting coordinates
        Predict angle, radius, action and segmentation from voi

        if action == 'move':
            Do update starting coordinates using angle and radius
        else if action == 'segment':
            Add segmentation of voi to scan_prediction given coordinates
            Move one axial slice in caudal direction
        else if action == 'stop':
            Stop inference procedure

return prediction
```
